# ﻿New faunistic records of chironomids and phantom midges (Diptera, Chironomidae and Chaoboridae) from Ukraine indicate recent climatic refugia in the Eastern Carpathians

**DOI:** 10.3897/zookeys.1211.125436

**Published:** 2024-09-09

**Authors:** Peter Bitušík, Milan Novikmec, Marek Svitok, Ladislav Hamerlík

**Affiliations:** 1 Faculty of Natural Sciences, Matej Bel University, Tajovského 40, SK-974 01 Banská Bystrica, Slovakia Matej Bel University Banská Bystrica Slovakia; 2 Faculty of Ecology and Environmental Sciences, Technical University in Zvolen, T. G. Masaryka 24, SK-960 53 Zvolen, Slovakia Technical University in Zvolen Zvolen Slovakia; 3 Department of Forest Ecology, Faculty of Forestry and Wood Sciences, Czech University of Life Sciences Prague, Kamýcka 129, CZ-165 21 Prague, Czech Republic Czech University of Life Sciences Prague Prague Czech Republic; 4 Institute of Zoology, Slovak Academy of Sciences, Dúbravská cesta 9, SK-845 06 Bratislava, Slovakia Institute of Zoology, Slovak Academy of Sciences Bratislava Slovakia

**Keywords:** climatic relicts, mountain lakes, pupal exuvia, submontane rivers

## Abstract

The aquatic insect fauna of the Eastern Carpathians is poorly known, especially in Ukraine. To address this knowledge gap, we conducted faunistic surveys of Chironomidae and Chaoboridae in 2018 and 2021. The study involved sampling of 11 watercourses and 10 mountain lakes situated in the Ukrainian part of the Eastern Carpathians. A total of 101 taxa were identified, including 40 chironomid species and one genus that have been recorded for the first time from Ukraine. The occurrence of one species previously considered as “doubtfully present” in Ukraine was confirmed by this study. One of the two identified phantom midge species, Chaoborus(s. str.)obscuripes (van der Wulp, 1859), is recorded for the first time from Ukraine. The most intriguing records are chironomid species Cricotopus(s. str.)beckeri Hirvenoja, 1973, *Eukiefferiellabedmari* Vilchez-Quero & Laville, 1987, and Pseudorthocladius(s. str.)berthelemyi Moubayed, 1990. These species have Mediterranean distribution and their occurrence in the Eastern Carpathians could be remains of once-widespread populations that currently survive in the Carpathian refugia due to adverse climatic conditions in the former distribution area. The high number of first records from a relatively small number of sites indicates a great gap in the knowledge of the Ukrainian chironomid fauna.

## ﻿Introduction

Chironomids are the most ubiquitous free-living holometabolous insects known from all zoogeographic regions, and all climatic zones from the tropics to the polar regions, including Antarctica (Ashe and O’Connor 2009). Recently, 7290 species belonging to nearly 440 genera and 11 subfamilies have been described worldwide (Ferrington 2007; Pape et al. 2011). In immature stages, most species inhabit various types of freshwaters although some species thrive in brackish water and intertidal pools, and few are truly marine. Finally, semi-terrestrial and fully terrestrial species are also known (Sæther et al. 2000). Among aquatic insects, Chironomidae is the most species-rich insect family found in freshwater ecosystems (Cranston 1995; Ferrington 2007). The species richness of the family recorded from only one stream locality is often astonishing. In some cases, the number of chironomid species recorded is higher than the diversity of all the other benthic macroinvertebrates (own observations).

Chironomidae can withstand an extremely wide range of environmental conditions in terms of water column depth, temperature, pH, dissolved oxygen, habitat drying and, finally, the gradient of human impacts such as pollution, habitat modification, and changes in watersheds (Ferrington 2007 and references therein). Consequently, chironomids have attracted the attention of researchers around the world as biological indicators for environmental impact assessments, ecosystem health, palaeolimnological reconstructions, and climate change (Resh and Rosenberg 2008; Eggermont and Heiri 2012; Nicacio and Juen 2015 and references therein). Compared to chironomids, the global diversity of phantom midges is low. The Chaoboridae family consists of about 51 extant species in six genera and two subfamilies (Borkent 2014). The immature stages usually live in standing waters, in some cases in small, temporary ponds. Larvae are predators and mostly planktonic; they are often considered keystone species that can eliminate or strongly suppress other invertebrates in the community (MacKay et al. 1990). Subfossil remains of the *Chaoborus* genus have been used in palaeoenvironmental research but also in contemporary ecological studies (e.g., Luoto and Nevalainen 2009; Tolonen et al. 2012). Despite the indisputable importance of both the chaoborids and chironomids and the rapid progress in the knowledge of their distribution, there are still areas that are “terra incognita”. The Eastern Carpathians are undoubtedly one of them.

Here, we present results from an ongoing faunistic inventory of chironomids and chaoborids from Ukrainian Carpathian Mountain lakes, which are supplemented by results from earlier investigations of the flowing waters in this territory.

## ﻿Materials and methods

### ﻿Study area

**Figure 1.**
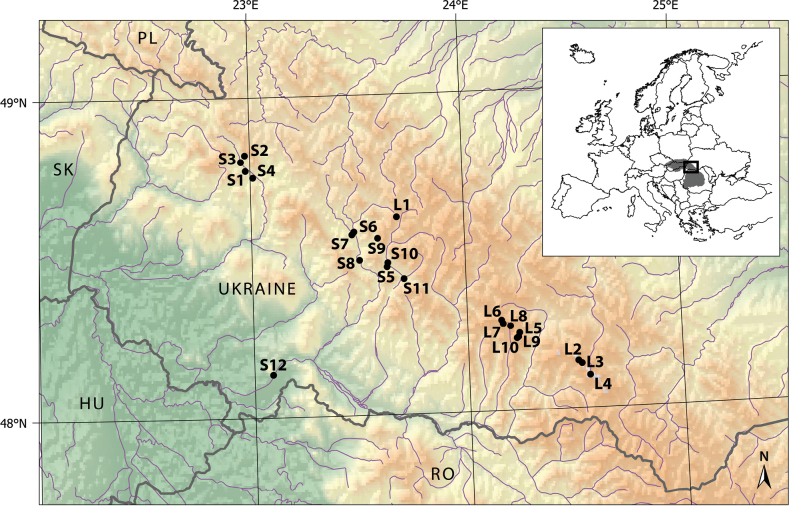
Map showing of sampling localities of Chironomidae and Chaoboridae in the Ukrainian part of the Eastern Carpathians. The site abbreviations correspond to the Table 1. Inset shows location of the investigated territory within the Carpathian Mountains (grey area).

This study was conducted in the Ukrainian Carpathians located in the northern part of the Eastern Carpathians, extending through the western part of Ukraine (Fig. 1). The total length of this mountain range is approximately 240 km with total area of ca 24,000 km^2^. The Ukrainian Carpathians are medium-high mountains with the highest elevation slightly exceeding 2000 m a.s.l. (Novikoff and Hurdu 2015; Vyshnevskyi and Donich 2021). The studied area is characterised by complex geology consisting mostly of flysch with different constituents. Only small areas are formed by limestone, shales, and volcanic rocks, predominantly andesites and gneisses (Ivanik et al. 2019). We sampled 11 streams and 10 mountain lakes (Table 1). Except for lakes Sinevir and Dragobratske located in deciduous and coniferous forests, respectively, all studied lakes are situated above 1500 m a.s.l., i.e., above formerly accepted climatic tree line (Kobiv 2017). The semi-natural meadows and pastures (so-called *polonynas* in the vernacular) dominate the catchment vegetation. The proportion of dwarf pine (*Pinusmugo*), dwarf juniper (Juniperuscommunissubsp.nana), green alder (*Alnusviridis*), and scattered Norway spruce (*Piceaabies*) is different in individual catchments.

**Table 1.** Basic characteristics of the sampling sites. Strahler stream order was estimated from Google Earth Pro. Stream width, depth and lake depth were estimated in the field. Lake area was derived from Google Earth Pro using Polygon tool.

Stream/Lake name	Code	Latitude, Longitude	Elevation (m)	Stream order	Av. width (m)	Av. depth (m)
Zhdenivka River	S1	48°46.29'N, 22°58.68'E	409	IV.	6	0.30
Nameless headwater stream	S2	48°49.19'N, 22°58.58'E	777	II.	2	0.15
Tributary of the Zhdenivka River	S3	48°47.98'N, 22°57.41'E	454	III.	3	0.20
Latoricja River	S4	48°45.00'N, 23°00.75'E	360	IV.	20	0.30
Gluhana peat bog channel	S5	48°27.94'N, 23°37.94'E	596	I.	0.4	0.10
Rika River (village Soimy)	S6	48°34.00'N, 23°28.53'E	449	IV.	35	0.15
Rypenka River (village Soimy)	S7	48°33.92'N, 23°28.29'E	448	IV.	25	0.30
Rika River (above Zaperedillia village)	S8	48°29.08'N, 23°30.24'E	382	V.	30	0.50
Nameless tributary of the Volovets River	S9	48°33.14'N, 23°35.51'E	725	II.	2	0.15
Tereblia River (village Sinevyr)	S10	48°28.25'N, 23°38.16'E	573	IV.	18	0.30
Sukhar brook (village Kolochava)	S11	48°25.40'N, 23°42.58'E	580	III.	10	0.30
Tisa River (Vinohradiv)	S12	48°08.06'N, 23°05.15'E	124	VII.	120	–
				Area (ha)	Max. depth (m)	
Sinevir Lake	L1	48°37.01'N, 23°41.04'E	989	4.20	22	
Brespo Lake	L2	48°08.62'N, 24°30.94'E	1627	0.03	0.4	
Bolotnoe ozerce	L3	48°08.55'N, 24°31.25'E	1695	0.01	0.9	
Brebeneskul Lake	L4	48°06.10'N, 24°33.74'E	1793	0.60	3.2	
Dragobratske Lake	L5	48°14.45'N, 24°14.45'E	1382	0.07	1.5	
Apshynets Lake	L6	48°16.91'N, 24°09.53'E	1491	2.97	1.8	
Geryshaska (Dohiaska) Lake	L7	48°16.25'N, 24°09.93'E	1585	2.58	2	
Kosivske Lake	L8	48°15.78'N, 24°11.96'E	1614	0.13	1	
Ivor Lake	L9	48°13.70'N, 24°14.10'E	1606	0.04	2	
Small Ivor Lake	L10	48°13.69'N, 24°14.07'E	1602	0.04	0.6	

All the studied flowing water sites except the Tisa River were located in the Carpathians. Most of the sampling sites were situated in valleys of the submontane belt up to 550–600 m a.s.l. (established according to climate and vegetation characteristics; Golubets 1977), only two sites (S2, S9) were situated in the lower montane belt above 700 m a.s.l. Stream bottom substrates were mostly dominated by cobbles with an admixture of finer components such as gravel and sand. The bottom habitats of both mountain brooks were more complex due to the presence of boulders and a considerable proportion of woody debris. Because of the bed surface roughness and shallow depth, turbulent flow is generally prevailing. Even though most sites were located near roads and often in settlements, the channels, banks, and riparian vegetation were visibly well preserved, without the signs of artificial modifications.

The above characteristics mostly do not apply to the Upper Tisa River. The river stretch close to the town of Vinohradiv flows in a lowland landscape (the Hungarian lowland ecoregion; Afanasyev et al. 2020). The floodplain is covered with alluvial forests containing willow and poplar. In the studied section, the low slope and high discharge altered the river channel morphology significantly, forming a high degree of channel sinuosity. The basic characteristics of the sampling sites are summarised in Table 1.

### ﻿Sampling and identification

Chironomids and chaoborids were collected during the sampling campaigns in May 2018 (flowing waters and Sinevir Lake) and in August 2021 (mountain lakes in the Chornohora and Svidovets Massifs). A hand net attached to a telescopic handle (mesh size 250 μm, frame diameter 25 cm) was used to skim the water surface and collect floating material along the shores of streams and lakes. In lakes, the material was collected at the leeward shore; in flowing waters, the floating material was collected along an ~ 100-meter-long stretch while moving upstream.

On the shore, the netted sample was placed in a labelled 100-ml plastic bottle and preserved with 75% ethanol. In the laboratory, the samples were placed in a Petri dish and all chironomid material was picked up under a stereomicroscope (7.5–50×). Sorted exuvia, pupae, and adults were mounted on microscopic slides and identified following the keys of Langton and Visser (2003), Ekrem (2004), Stur and Ekrem (2006), and Langton et al. (2013) for pupal exuvia and Langton and Pinder (2007a, 2007b) for adults under high magnification (400×) using phase contrast. Chaoborid larvae, which were collected accidentally during the chironomid sampling of lakes, were identified using Parma (1969) and Sæther (1997).

The nomenclature and distribution of species are consistent with Fauna Europaea (de Jong 2016), and Ashe and O’Connor (2009, 2012). Voucher specimens are deposited in the collections of the Dept. of Biology and Environmental Studies, Faculty of Natural Sciences, Matej Bel University in Banská Bystrica.

## ﻿Results and discussion

A total of 2088 specimens were collected and identified as 99 chironomid species/taxa (belonging to 43 genera from 5 subfamilies) and two chaoborid species of the same subfamily and genus. Altogether, 40 species and one genus of Chironomidae, and one species of Chaoboridae were recorded for the first time in Ukraine. The occurrence of one chironomid species, *Nilotanypusdubius* (Meigen, 1804), previously considered as “doubtfully present” in Ukraine (Spies and Sæther 2013) was finally confirmed. A list of all species/ taxa recorded is given below (sampling site codes refer to Table 1; **Pe** after the genus name refers to a morphotype not associated with an adult by Langton (1991); # – previously doubtfully present in Ukraine, * – first record of species/ genus from Ukraine). For detailed information on the abundance and life stages of collected specimens see Supplementary file 1.

### ﻿CHIRONOMIDAE

**Tanypodinae**

Procladius (Holotanypus) choreus (Meigen, 1804): L5, L6, L9

*Nilotanypusdubius* (Meigen, 1804)#: S1, S6, S7, S8

*Thienemannimyiacarnea* (Fabricius, 1805)*: S7

*Zavrelimyiabarbatipes* (Kieffer, 1911): L6

**Diamesinae**

Diamesa (Diamesa) cinerella Meigen, 1835*: S8

Diamesa (Diamesa) cf. tonsa (Haliday, 1856): S6

Diamesa (Diamesa) vaillanti Serra-Tosio, 1972*: S7

*Potthastia* Pe1 Langton, 1991: S4, S7, S8

*Sympotthastiamacrocera* Serra-Tosio, 1973*: S6

**Prodiamesinae**

*Prodiamesaolivacea* (Meigen, 1818): L1

**Orthocladiinae**

*Brilliabifida* (Kieffer, 1909): S3

*Brilliaflavifrons* (Johannsen, 1905)*: S7

*Corynoneuraceltica* Edwards, 1924*: S1, S3, S10

Corynoneuracf.scutellata Winnertz, 1846: L1, L6

*Corynoneura* Pe2a Langton, 1991: S1, S4, S5, S6, S7, S8

*Corynoneura* Pe4 Langton, 1991: S1

Cricotopus (Cricotopus) annulator Goetghebuer, 1927: S1, S4, S5, S6, S7

Cricotopus (Cricotopus) beckeri Hirvenoja, 1973*: S4, S6, S8

Cricotopus (Cricotopus) curtus Hirvenoja, 1973*: S6

Cricotopus (Cricotopus) fuscus (Kieffer, 1909): S1, S11

Cricotopus (Cricotopus) pallidipes Edwards, 1929*: S8

Cricotopus (Cricotopus) similis Goetghebuer, 1921*: S1, S7, S8

Cricotopus (Cricotopus) tremulus (Linnaeus, 1758)*: S1, S3, S6, S8

Cricotopus (Cricotopus) trifascia Edwards, 1929: S4, S11

Cricotopus (Cricotopus) vierriensis Goetghebuer, 1935: S1, S4, S6, S7, S8

*Cricotopus* Pe 17 Langton, 1991: S4, S8

Cricotopus (Isocladius) reversus Hirvenoja, 1973*: L1

Cricotopus (Isocladius) sylvestris (Fabricius, 1794): L6, L7

Cricotopus (Isocladius) Pe 5 Langton, 1991: L6, L7

Cricotopus (Paratrichocladius) rufiventris (Meigen, 1830): S4, S7, S10

*Eukiefferiellabedmari* Vilchez-Quero & Laville, 1987*: S4

*Eukiefferiellabrevicalcar* (Kieffer, 1911): S2, S3

*Eukiefferiellaclypeata* (Thienemann, 1919)*: S6, S7, S11

*Eukiefferiellacoerulescens* (Kieffer, 1926): S3, S8

*Eukiefferielladevonica* (Edwards, 1929): S9

*Eukiefferiellafuldensis* Lehmann, 1972: S10

*Eukiefferiellailkleyensis* (Edwards, 1929): S4, S6, S7, S8, S10

*Euryhapsis* Pe1 Langton, 1991: S11

*Heleniellaserratosioi* Ringe, 1976*: S1, S4, S6, S7, S8, S9, S10, S11

*Krenosmittiaboreoalpina* (Goetghebuer, 1944)*: S1, S3, S6, S7, S8, S10

*Krenosmittiacamptophleps* (Edwards, 1929)*: S10

Limnophyescf.asquamatus Andersen, 1937: L3

Nanocladius (Nanocladius) parvulus (Kieffer, 1909): S3, S4, S6, S8

Nanocladius (Nanocladius) rectinervis (Kieffer, 1911)*: S6, S7, S8, S10, S11

Orthocladius (Orthocladius) dentifer Brundin, 1947: L7

Orthocladius (Orthocladius) excavatus Brundin, 1947*: S7, S8, S11

Orthocladius (Orthocladius) oblidens (Walker, 1856)*: S11

Orthocladius (Orthocladius) pedestris Kieffer, 1909*: S1, S3, S4, S7, S8, S11

Orthocladius (Orthocladius) rivinus Potthast, 1914*: S3

Orthocladius (Orthocladius) rubicundus (Meigen, 1818): S1, S3, S4, S7, S8, S10

Orthocladius (Euorthocladius) ashei Soponis, 1990*: S4, S7, S8, S11

Orthocladius (Euorthocladius) rivicola Kieffer, 1911: S3, S6, S8, S10, S11

Orthocladius (Euorthocladius) rivulorum Kieffer, 1909: S4, S6, S7, S8

*Paracricotopusniger* (Kieffer, 1913)*: S4, S6, S7, S8, S11

*Parakiefferiellabathophila* (Kieffer, 1912)*: S4, S7, S8

*Parametriocnemusstylatus* (Spaerck, 1923): S1, S3, S4, S6, S7, S8, S9, S10, S11

Psectrocladius (Psectrocladius) limbatellus (Holmgren, 1869): S11

Psectrocladius (Psectrocladius) oligosetus Wuelker, 1956: L2, L3, L9, L10

Psectrocladius (Psectrocladius) schlienzi Wuelker, 1956*: L1

Pseudorthocladius (Pseudorthocladius) berthelemyi Moubayed, 1990*: S6

Rheocricotopus (Psilocricotopus) chalybeatus (Edwards, 1929): S1, S4, S6, S7, S8

Rheocricotopus (Rheocricotopus) fuscipes (Kieffer, 1909): S1, S3, S7, S8

*Rheosmittiaspinicornis* (Brundin, 1956)*: S2, S3, S10

*Symbiocladiusrhithrogenae* (Zavrel, 1924): S11

*Synorthocladiussemivirens* (Kieffer, 1909): S3, S10

*Thienemanniellamajuscula* (Edwards, 1924): S1

*Thienemanniella* Pe 1b Langton, 1991: S3

*Thienemanniella* Pe 2 Langton, 1991: S10

*Tveteniaverralli* (Edwards, 1929)*: S6

**Chironominae**

*Benthalia* sp.: L7

*Demicryptochironomus* Pe1 Langton, 1991: S11

*Microtendipeschloris* (Meigen, 1818): S5, S7

*Microtendipespedellus* (De Geer, 1776): L6

*Paracladopelmamikianum* (Goetghebuer, 1937)*: S6, S11

*Phaenopsectraflavipes* (Meigen, 1818): S5, L1, L2, L6, L7

Polypedilum (Polypedilum) albicorne (Meigen, 1838)*: S4, S6, S7, S11

Polypedilum (Polypedilum) laetum (Meigen, 1818): S11

Polypedilum (Polypedilum) nubeculosum (Meigen, 1804): S11

Polypedilum (Pentapedilum) sordens (van der Wulp, 1875): S7

Polypedilum (Pentapedilum) cf. uncinatum (Goetghebuer, 1921): L2, L5, L7

Polypedilum (Tripodura) cf. apfelbecki (Strobl, 1900): S6

Polypedilum (Uresipedilum) convictum (Walker, 1856): S4, S11

Cladotanytarsus (Cladotanytarsus) atridorsum Kieffer, 1924: L6, L7, L8

Cladotanytarsus (Cladotanytarsus) vanderwulpi (Edwards, 1929): S6, S7, S8

*Micropsectraatrofasciata* (Kieffer, 1911): S6, S7

*Micropsectralindrothi* Goetghebuer, 1931*: L7

*Micropsectralindebergi* Saewedal, 1976/ *insignilobus* Kieffer, 1924: S5

*Neozavrelia* Pe1 Langton, 1991*: S1, S4, S5, S6, S7, S8

*Paratanytarsusaustriacus* (Kieffer, 1924): L6

*Paratanytarsusdissimilis* (Johannsen, 1905)*: S5

*Paratanytarsuslaccophilus* (Edwards, 1929): L2, L5, L7, L8, L9, L10

*Rheotanytarsuspentapoda* (Kieffer, 1909)*: S6, S7

*Rheotanytarsusrhenanus* Klink, 1983*: S1, S6, S7

*Stempellinellaflavidula* (Edwards, 1929)*: S8

*Tanytarsusaberrans* Lindeberg, 1970*: L6

*Tanytarsusdebilis* (Meigen, 1830)*: L5, L7

*Tanytarsusgregarius* Kieffer, 1909: L2, L4

*Tanytarsusheusdensis* Goetghebuer, 1923*: S6, S7

*Virgatanytarsus* Pe1 Langton, 1991: S4

### ﻿CHAOBORIDAE

**Chaoborinae**

Chaoborus (Chaoborus) crystallinus (De Geer, 1776): L3

Chaoborus (Chaoborus) obscuripes (van der Wulp, 1859)*: L7

Of the 99 recorded chironomid taxa, 22 were found exclusively in lakes, and 20 of them only in alpine lakes. Fourteen lacustrine species/ taxa (i.e., 70%) were also found in lakes during our previous research (Bitušík et al. 2020) indicating a similar species composition of all the studied lakes. Phantom midges were not deliberately targeted since the larvae recorded were caught by chance while collecting pupal exuvia. However, one of the species recorded, Chaoborus(s. str.)obscuripes (van der Wulp, 1859), appears to be the first record in the Ukrainian Carpathian alpine lakes. Most chironomid species have been found in flowing waters. Overall, they are considered typical for streams and rivers of Western and Central Europe (e.g., Caspers 1991; Laville and Vinçon 1991; Ruse 1995; Orendt 2002a, 2002b; Schöll and Haybach 2004; Bitušík et al. 2006; Calle-Martínez and Casas 2006; Prat et al. 2016 and citation therein). Because most of the newly recorded species are widespread in stagnant and flowing waters in Europe and the Palaearctic Region, and do not increase our knowledge on their ecology, only species with restricted distributions and rarely collected elsewhere are discussed further in more detail. Regarding the common species not discussed further in the text, their geographical distribution is documented in Ashe and O’Connor (2009, 2012) and de Jong (2016), while their ecology is summarised in Moller Pillot (2009, 2013) and Vallenduuk and Moller Pillot (2007).

### ﻿Family Chironomidae

**Subfamily Diamesinae**

**Tribe Diamesini**

#### ﻿ Diamesa (Diamesa) vaillanti

Taxon classificationAnimaliaDipteraChironomidae

Serra-Tosio, 1972

9D59C8A4-0647-525C-BB3B-DAC77B3A796E

##### Material examined.

1 pupal exuvium, Rypenka river (S7), 7 May 2018.

##### Distribution.

Palaearctic: Germany, Switzerland, France, Italy, Austria, Slovakia, Poland, Russia, Spain, Turkey, Morocco (Ashe and O’Connor 2009), and Azerbaijan (Kownacki 1985).

##### Habitat.

Rheophilic species inhabiting high-altitude springs, streams (including glacier-fed), and rivers with rocky bottoms, but also alpine lakes (Bitušík 2004; Lencioni et al. 2011; Rossaro and Lencioni 2015). The species is cold-stenothermal (e.g., Rossaro 1991) although the findings from streams in the High Atlas indicate that it can tolerate relatively high temperatures of up to 22 °C (Azzouzi et al. 1992).

##### Remarks.

The occurrence of the species is limited to waters at high altitudes. Since our record is from 448 m a.s.l., our finding is exceptional.

#### ﻿ Sympotthastia macrocera

Taxon classificationAnimaliaDipteraChironomidae

Serra-Tosio, 1973

32C3DBA5-5039-5783-AAB7-8E795F401387

##### Material examined.

2 pupal exuvia, Rika River (S6) in Soimy village, 7 May 2018.

##### Distribution.

Palaearctic. For a long time, known only from Western Europe (France, Germany; Ashe and O’Connor 2009). More recent data comes from the Drava River in Croatia (Kresonja 2018). Our record is evidence for the current easternmost occurrence in Europe, but there are indications that the distribution of this species may extend as far as the Ural Mountains (Krasheninnikov 2012).

##### Habitat.

Generally, larvae of the genus *Sympotthastia* inhabit cold running waters and springs (Sæther and Andersen 2013). A few data indicate that *S.macrocera* is probably a rheophilic species.

##### Remarks.

*Sympotthastiamacrocera* appears to be a relatively rare species with little known ecology.

### ﻿Subfamily Orthocladiinae

#### ﻿ Cricotopus (Cricotopus) beckeri

Taxon classificationAnimaliaDipteraChironomidae

Hirvenoja, 1973

563DDDB4-5632-5135-8F99-74AA007FA8AF

##### Material examined.

1 pupal exuvium, Latoricja River (S4), 5 May 2018; 1 pupal exuvium, Rika River (S6); 2 pupal exuvia, Rika River (S8) above Zaperedillia village, 7 May 2018.

##### Distribution.

Palaearctic. France, Spain, Greece, Madeira, Corsica, Turkey, Algeria, Morocco, Slovakia. Its questionable occurrence in Finland (Ashe and O’Connor 2012) was not accepted later Paasivirta (2014).

##### Habitat.

Principally inhabits the rhithral zone of streams at lower altitudes (Hirvenoja and Moubayed 1989; Kettani and Langton 2012; Moubayed-Breil and Ashe 2016).

##### Remarks.

*Cricotopusbeckeri* has been considered an exclusively Mediterranean species (Laville and Reiss 1992). Our record is the second reliable finding in the Carpathians (Bitušík and Langton 1994) far from its continuous distribution. We assume that the isolated populations in the Carpathians could be remnants of once widespread populations, which currently survive in refugia due to adverse climatic conditions. Thus, they could be considered a climatic relict, as customary for some plant species (Molnár et al. 2017). Interestingly, Reiss (1986) already hypothesised that the extra-Mediterranean occurrence of another Mediterranean chironomid, *Paratanytarsusmediterraneus* Reiss, 1981, could have a relict character in the Middle Rhine.

#### ﻿ Cricotopus (Cricotopus) pallidipes

Taxon classificationAnimaliaDipteraChironomidae

Edwards, 1929

755D51E5-0D69-5A2B-BDB0-8011CEB5801D

##### Material examined.

1 pupal exuvium, Rika River (S8), 7 May 2018.

##### Distribution.

Palaearctic. Finland, Norway, France, Portugal, Spain, Germany, Great Britain, Ireland, Romania, Hungary, Russia, Lebanon, and Morocco (Cobo et al. 2002; Soriano and Cobo 2006; Móra et al. 2006; Ashe and O’Connor 2012).

##### Habitat.

The ecological requirements of this species are still unclear. It has been found in flowing and stagnant waters in cold climatic zones (Aagaard et al. 1997; Pozdeev 2012) to warm rivers, canals, lakes, and marshes in central and southern Europe, and North Africa (e.g., Laville and Tourenq 1968; Móra et al. 2006; Abbou and Fahde 2017; Moubayed et al. 2019). French authors (Tourenq 1976; Moubayed et al. 2019) consider it a lacustrine species, tolerant of low oxygen content.

##### Remarks.

Currently known only from few European countries. It does not seem to be abundant anywhere. In Bavaria and the Sauerland Mountains (Germany), it is listed among possibly endangered species; however, its status is unknown (Orendt and Reiff 2003; Dittmar 2012).

#### ﻿ Eukiefferiella bedmari

Taxon classificationAnimaliaDipteraChironomidae

Vilchez-Quero & Laville, 1987

C916796D-EB5B-5760-AB6D-2E38A1250C61

##### Material examined.

3 pupal exuvia, Latoricja River (S4), 5 May 2018.

##### Distribution.

Palaearctic. France, Spain, Greece, Corsica, Turkey, Lebanon, Algeria, and Morocco (Ashe and O’Connor 2012).

##### Habitat.

Streams and rivers (Laville and Langton 2002; Chaib et al. 2013; Moubayed-Breil and Ashe 2016).

##### Remarks.

*E.bedmari* is a circum-mediterranean faunistic element (Laville and Reiss 1992; Moubayed-Breil 2008). Our unexpected extra-Mediterranean finding suggests the relict character of its population in the Carpathians (see comments to *Cricotopusbeckeri*).

#### ﻿ Orthocladius (Orthocladius) rivinus

Taxon classificationAnimaliaDipteraChironomidae

Potthast, 1914

6DF9222C-78D4-53BA-82C9-22D717919E0B

##### Material examined.

1 pupal exuvium, left-hand tributary of Zhdenivka River (S3), 7 May 2018.

##### Distribution.

Palaearctic. Norway, Great Britain, Ireland, Austria, Slovakia, Hungary, Poland, Belarus, Germany, Switzerland, France, Italy, Spain, Canary Islands, and Portugal (Cobo et al. 2002; Ashe and O’Connor 2012; Moller Pillot 2013; Móra et al. 2013; Sołtys-Lelek et al. 2014).

##### Habitat.

Rheophilic species inhabiting springs and flowing waters from small streams to large rivers, although it has been reported also from lakes (Langton and Visser 2003). Rossaro et al. (2003) underline its preference for cold waters, but some findings question this (e.g., Langton and Orendt 1996; Móra et al. 2013).

##### Remarks.

The species is known from a few European countries and is generally considered rare. Like the ambiguous data on its ecology, this may also be the result of misidentification.

#### ﻿ Psectrocladius (Psectrocladius) schlienzi

Taxon classificationAnimaliaDipteraChironomidae

Wuelker, 1956

1ED69ECE-E09E-5E09-88BA-2A1EB3B2DCAD

##### Material examined.

1 pupal exuvium, Lake Sinevir (L1), 7 May 2018.

##### Distribution.

Palaearctic? In addition to some European countries (Austria, Denmark, Finland, Germany, Great Britain, Italy, Moldova, Netherlands, Norway, Portugal, Slovakia, Czech Republic, Spain, Sweden, Switzerland; Ashe and O’Connor 2012; Syrovátka and Langton 2015), it was also recorded in Mongolia (Hayford 2005). However, the species may have a Holarctic distribution, provided that its presence in North America can be confirmed (Sealock and Ferrington 2008). Baranov et al. (2024) found a species resembling *P.schlienzi* in Namibia, but it is possible that the specimen belongs to a yet undescribed species of *Psectrocladius* related to *P.schlienzi*.

##### Habitat.

Different types of stagnant waters from lakes to pools. For example, the only record from the Carpathians comes from a shallow pond in an exploited part of an alkaline fen (Bitušík and Illéšová 1998). Its occurrence in slowly flowing waters is exceptional (de Beauvesère-Storm and Tempelman 2009).

##### Remarks.

The records are scattered across Europe, and it seems that the species is not abundant anywhere (Moller Pillot 2013).

#### ﻿ Pseudorthocladius (Pseudorthocladius) berthelemyi

Taxon classificationAnimaliaDipteraChironomidae

Moubayed, 1990

CAC4437C-BCAD-5948-A382-A40E87AE9C11

##### Material examined.

11 pupal exuvia, Rika River (S6), 7 May 2018.

##### Distribution.

Palaearctic. Austria, Bulgaria, Corsica, France, Germany, Portugal, Slovakia, Spain, Turkey, and Morocco (Ashe and O’Connor 2012).

##### Habitat.

Mountain streams and rivers with stony bottoms. The species is rheophilic, cold-stenothermal with high demand for dissolved oxygen (Martínez et al 1995; Moubayed-Breil et al. 2012). It can also inhabit hygropetric sites (Moubayed-Breil 2008).

##### Remarks.

The species is considered a Mediterranean element (Moubayed-Breil 2008) with an originally circum-mediterranean distribution (Laville and Langton 2002). The extra-Mediterranean occurrence in more northerly countries indicates its relict character (see comments to *Cricotopusbeckeri*).

### ﻿Subfamily Chironominae

**Tribe Chironomini**

#### ﻿ Paracladopelma mikianum

Taxon classificationAnimaliaDipteraChironomidae

(Goetghebuer, 1937)

278DEA6F-863E-551C-912B-77AD3E9B7667

##### Material examined.

2 pupal exuvia, Rika River (S6), 7 May 2018; 1 pupal exuvium, Tisa River (S12), 8 May 2018.

##### Distribution.

Palaearctic. The species was recorded only from a few countries in Europe (e.g., Spain, Hungary, Slovakia, Portugal, France, Germany, Romania) and North Africa (Morocco, Lebanon).

##### Habitat.

It is a rheophilic species inhabiting fast-flowing streams and rivers (Moller Pillot 2009). Although Calle-Martínez and Casas (2006) listed the species in a chironomid community usually associated with low-temperature or torrential mountain streams, our finding from Tisa River and other records from large lowland rivers (Gandouin et al. 2006; Klink 2010) indicate that the species does not have as strict cold temperature preferences as though previously (Ringe 1974).

##### Remarks.

Laville and Vinçon (1986) considered the species to be a Mediterranean-Palaearctic element whose northern limit is located in the Pyrenees, the Alps, and the Carpathians.

### ﻿Tribe Tanytarsini

#### ﻿ Neozavrelia

Taxon classificationAnimaliaDipteraChironomidae

Goetghebuer, 1941

68F5E2B9-E18A-5371-9F33-D24826E917AD

##### Material examined.

11 pupal exuvia, Zhdenivka River (S1), 5 May 2018; 17 pupal exuvia, Latoricja River (S4), 5 May 2018; 1 pupal exuvium, Rika River (S6), 7 May 2018; 18 pupal exuvia Rika River (S8), 7 May 2018; 19 pupal exuvium, Rypenka River (S7), 7 May 2018; 1 pupal exuvium, channel at Gluhana peat bog (S5), 7 May 2018.

##### Distribution.

Species-rich genus (38 valid species, de Jong 2016) with a worldwide distribution except for Africa and Neotropics (Epler et al. 2013). Five species have been recorded in Europe, three of which are reliably confirmed in the Carpathians: *N.improvisa* Fittkau, 1954, *N.luteola* Goetghebuer, 1941 (Giłka 2007), and *N.cuneipennis* (Edwards, 1929) (Tatole 2023).

##### Habitat.

Larvae of *Neozavrelia* inhabit streams, rivers, lakes, and ponds in peat bogs; they are also known from hygropetric sites, and one species lives in a hot spring (Epler et al. 2013).

##### Remarks.

Except for *N.cuneipennis* (= *N.longappendiculata* Albu, 1980), the morphological characteristics of the pupae do not yet allow for distinguishing the European species (Langton and Visser 2003). The morphotype *Neozavrelia* Pe1 Langton, 1991 includes four species: *N.bernensis* Reiss, 1968, *N.fuldensis* Fittkau, 1954, *N.improvisa*, and *N.luteola*.

### ﻿Family Chaoboridae

#### ﻿ Chaoborus (Chaoborus) obscuripes

Taxon classificationAnimaliaDipteraChaoboridae

(van der Wulp, 1859)

84792A91-C142-513D-8679-583FC8295032

##### Material examined.

1 larva, Lake Geryshaska (L7), 15 September 2021.

##### Distribution.

Palaearctic. The species is widespread mainly in Northern and Western Europe, but also in Poland and the European part of Russia (Borkent 1981; de Jong 2016).

##### Habitat.

Small, shallow nutrient-poor, meso- and polyhumic ponds with pH 4.5–5.5 (Nilssen 1974; Joniak and Domek 2006; Kuper and Verberk 2011), often fishless. Larger larvae with darker pigmentation are more sensitive to visually dependent predators (Stenson 1981).

##### Remarks.

The species seems to occur sporadically and mostly in small numbers (Borkent 1981), which is probably related to its ecological requirements for water chemistry and the absence of fish.

The first annotated checklist of Ukrainian Chironomidae consists of 302 species (Baranov 2011a). However, this list requires revision because it contains invalid species identified solely on the basis of larvae using outdated identification keys. In recent decades, the study of taxonomy, ecology, and biogeography of chironomids in Ukraine has intensified (Baranov 2011b, 2013, 2014; Baranov and Przhiboro 2014; Baranov and Ferrington 2013; Moubayed-Breil and Baranov 2018; Didenko et al. 2021). Our survey revealed a significant gap in the taxonomic knowledge of Ukrainian chironomids. The high number of new records suggests that the chironomid fauna, especially from flowing waters is far from being fully discovered. Undoubtedly, it is necessary to continue the study of the chironomid fauna of the Eastern Carpathians. Particularly, the collection of the pupal exuvia could be a very useful tool in studying species richness, ecology, and distribution, but also for water quality assessment and monitoring purposes.

## Supplementary Material

XML Treatment for Diamesa (Diamesa) vaillanti

XML Treatment for Sympotthastia macrocera

XML Treatment for Cricotopus (Cricotopus) beckeri

XML Treatment for Cricotopus (Cricotopus) pallidipes

XML Treatment for Eukiefferiella bedmari

XML Treatment for Orthocladius (Orthocladius) rivinus

XML Treatment for Psectrocladius (Psectrocladius) schlienzi

XML Treatment for Pseudorthocladius (Pseudorthocladius) berthelemyi

XML Treatment for Paracladopelma mikianum

XML Treatment for Neozavrelia

XML Treatment for Chaoborus (Chaoborus) obscuripes

## ﻿Additional information

### Author ORCIDs

Milan Novikmec https://orcid.org/0000-0002-5192-4575

Marek Svitok https://orcid.org/0000-0003-2710-8102

### Data availability

All of the data that support the findings of this study are available in the main text or Supplementary Information.

## Supplementary materials

Supplementary material 1 Checklist of chironomid and chaoborid taxa recorded in studied streams and lakes of the Ukrainian part of the Eastern Carpathians Data type xlsx https://binary.pensoft.net/file/1130307This dataset is made available under the Open Database License (http://opendatacommons.org/licenses/odbl/1.0/). The Open Database License (ODbL) is a license agreement intended to allow users to freely share, modify, and use this Dataset while maintaining this same freedom for others, provided that the original source and author(s) are credited.Peter Bitušík, Milan Novikmec, Marek Svitok, Ladislav Hamerlík
